# Detection and variant characterization of lumpy skin disease virus from dairy cattle in India

**DOI:** 10.1093/ve/veaf090

**Published:** 2025-11-20

**Authors:** Manali Bajpai, Ajinkya Khilari, Bhagyashree Likhitkar, Pankaj Musale, Santoshkumar Jadhav, Velu Dhanikachalam, Payal Kakramkar, Kaustubh Bhave, Marimuthu Swaminathan, Sachin Joshi, Dhanasekaran Shanmugam

**Affiliations:** Biochemical Sciences Division, CSIR- National Chemical Laboratory, Dr. Homi Bhabha Road, Pune, Maharashtra 411008, India; Academy of Scientific and Innovative Research (AcSIR), Sector 19, Kamla Nehru Nagar, Ghaziabad, Uttar Pradesh 201002, India; Biochemical Sciences Division, CSIR- National Chemical Laboratory, Dr. Homi Bhabha Road, Pune, Maharashtra 411008, India; Academy of Scientific and Innovative Research (AcSIR), Sector 19, Kamla Nehru Nagar, Ghaziabad, Uttar Pradesh 201002, India; Biochemical Sciences Division, CSIR- National Chemical Laboratory, Dr. Homi Bhabha Road, Pune, Maharashtra 411008, India; Academy of Scientific and Innovative Research (AcSIR), Sector 19, Kamla Nehru Nagar, Ghaziabad, Uttar Pradesh 201002, India; Biochemical Sciences Division, CSIR- National Chemical Laboratory, Dr. Homi Bhabha Road, Pune, Maharashtra 411008, India; Academy of Scientific and Innovative Research (AcSIR), Sector 19, Kamla Nehru Nagar, Ghaziabad, Uttar Pradesh 201002, India; BAIF Development Research Foundation, BAIF Road, Uruli Kanchan, Pune, Maharashtra 412202, India; BAIF Development Research Foundation, BAIF Road, Uruli Kanchan, Pune, Maharashtra 412202, India; BAIF Development Research Foundation, BAIF Road, Uruli Kanchan, Pune, Maharashtra 412202, India; BAIF Development Research Foundation, BAIF Road, Uruli Kanchan, Pune, Maharashtra 412202, India; BAIF Development Research Foundation, BAIF Road, Uruli Kanchan, Pune, Maharashtra 412202, India; Freelance Consultant, Central Research Station, BAIF Development Research Foundation, BAIF Road, Uruli Kanchan, Pune - 412202, India; BAIF Development Research Foundation, BAIF Road, Uruli Kanchan, Pune, Maharashtra 412202, India; Biochemical Sciences Division, CSIR- National Chemical Laboratory, Dr. Homi Bhabha Road, Pune, Maharashtra 411008, India; Academy of Scientific and Innovative Research (AcSIR), Sector 19, Kamla Nehru Nagar, Ghaziabad, Uttar Pradesh 201002, India

**Keywords:** lumpy skin disease, multiplexed nested PCR, virus surveillance, genotyping by sequencing, Oxford Nanopore technology

## Abstract

The spread of a severe and often fatal form of lumpy skin disease (LSD) in cattle and water buffaloes has caused widespread mortality and morbidity of these animals in India. To track and understand the genetic changes occurring in the virus and to enable routine surveillance of the virus, multiplexed polymerase chain reaction (PCR) and sequencing methods were developed and validated in this study. Multiplexed nested PCR for LSD virus (LSDV) detection was optimized using skin lesion swabs and nasal samples collected from symptomatic and asymptomatic animals. For genotyping, overlapping PCRs to amplify the entire LSDV genome were developed and tested on field samples collected from the Maharashtra and Odisha states of India. Analysis of LSDV genomes from 41 field samples collected in 2022 and 2023 revealed the presence of highly conserved novel mutations. Phylogenetic analysis shows that a distinct genotype of LSDV has spread across India, which warrants genomic surveillance of the virus in the coming years to track the evolution and transmission of the virus. The non-invasive sample collection, detection, and genotyping methods described in this study can facilitate large-scale surveillance of LSDV in dairy animals.

## Introduction

Bovine lumpy skin disease (LSD) is caused by the LSD virus (LSDV), a poxvirus of the Capripoxvirus genus and Poxviridae family (Al-Salihi 2014, Mulatu and Feyisa 2018). The genus also includes the goat pox and sheep pox viruses, and together they cause a significant health impact on domestic ruminants (Tuppurainen et al. 2021b). LSDV is an enveloped, linear double-stranded DNA virus, with a ~151 kb genome containing 2.4 kb inverted terminal repeats (ITRs) at the ends (Putty et al. 2023). The viral genome is annotated with 156 putative genes, encoding structural and non-structural proteins (Gupta et al. 2020), which are highly conserved and antigenically similar in the Capripoxvirus genus (Fawzi et al. 2022).

LSD is a host-specific transboundary disease affecting cattle (*Bos taurus*, *Bos indicus*) and water buffalo (*Bubalus bubalis*) (Moudgil et al. 2024), and is listed in the WOAH (World Organization for Animal Health) website as a notifiable disease (https://www.woah.org/en/disease/lumpy-skin-disease/). Although experimental infections in impala and giraffe have shown clinical symptoms, there is no evidence of natural infection in these animals (Guyassa 2022). Recent reports of LSDV in *Gazella bennettii* in Rajasthan, India, suggest potential host range expansion, posing a significant threat to both domestic and wildlife species (Sudhakar et al. 2023). Although LSD is classified as non-zoonotic, transmission to humans cannot be ruled out. LSDV was detected in samples from human subjects suspected of infection in a study by the Animal Health Research Institute (AHRI), Egypt (Kamal 2019, Pal and Gutama 2023).

LSDV transmission can occur *via* ticks (*Rhipicephalus* and *Amblyomma* species), biting flies (*Stomoxys calcitrans*), and mosquitoes (*Aedes aegypti*) (Gupta et al. 2020) and is facilitated by abiotic factors such as animal trade, introduction of animals sourced from outside the herd, shared feed or water sources, vertical transmission, and nursing from infected udders (Thota and Manda 2023). LSDV can remain viable in the environment under optimal conditions, which can facilitate transmission of the virus and spread of disease (Gupta et al. 2020). While cattle of all ages, different breeds, and both sexes are susceptible to LSDV, lactating cows, young calves, and underweight cattle show higher vulnerability (Şevik et al. 2016). Clinical symptoms of LSD, including the prominent skin nodules, are well documented (Kumar et al. 2021, Hasib et al. 2021, Tuppurainen et al. 2021a) and disease morbidity ranges from 3% to 85% (Leliso et al. 2021), while mortality is reported at < 10% (Mathivanan et al. 2023).

LSDV was initially reported from Zambia and South Africa in the 1920s and is now known to be prevalent across Africa, the Middle East, Europe, and Asia (Paungpin et al. 2022). In India, LSDV was first reported in 2019 to affect livestock of marginal farmers in Odisha state (Sudhakar et al. 2020) with 7% morbidity and no mortality (Bhatt et al. 2023) However, by 2020, LSDV infections turned fatal, heavily impacting the northwest regions of Rajasthan, Gujarat, and Maharashtra states (Reddy et al. 2023) and by 2022, ~2.5 million cases and over 100 000 cattle deaths were recorded in India (Mathivanan et al. 2023). As India has the world’s largest dairy cattle population (https://dahd.gov.in), the economic impact of LSD on its dairy industry is substantial. With the rapid spread of LSDV across India and the appearance of new genetic variations in local isolates, there is a need for sensitive diagnostic tools and genomic surveillance protocols. In this study, we have developed methods that can help address this requirement: a multiplexed nested PCR method designed for sensitive detection of LSDV and a whole-genome sequencing method for genotyping the virus. We then applied these methods to field samples collected from Maharashtra and Odisha, generating high-quality genome data that allowed us to perform detailed phylogenomic analysis. Genome sequencing of LSDV isolates from India in this and previous studies (Bhatt et al. 2023, Yadav et al. 2024) has revealed novel mutations compared to earlier reported LSDV sequences from other countries. Therefore, sustained genomic surveillance of LSDV from across India is essential (Putty et al. 2023) to track the genetic variations that can occur in future. By combining a practical diagnostic assay with genomic characterization, we aim to provide tools that support early diagnosis, improve monitoring of viral evolution, and strengthen disease surveillance strategies for LSDV in India.

## Materials and methods

### Sample collection and DNA extraction

**Figure 1:**
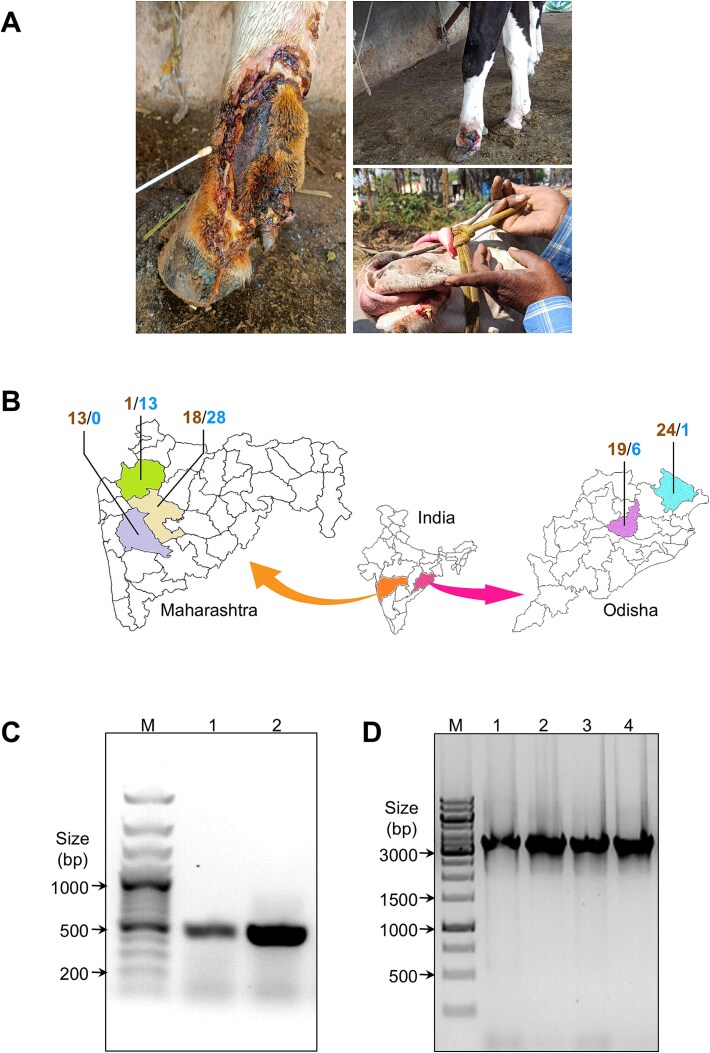
LSDV sampling and detection showing: (A) Photographs of dairy cattle showing the collection of samples from skin scabs and lesions on limbs and other parts. (B) Cartographic representation of the number of skin scab swab (mocha) and nasal swab (sky) samples collected from different districts of Maharashtra (Nashik, green; Ahmednagar, tan; Pune, purple) and Odisha (Mayurbhanj, cyan; Angul, pink). (C) Amplification of ~500 bp fragment obtained from the multiplexed nested PCR reaction. Lane M—100 bp DNA size ladder; lane 1 & lane 2—representative samples showing positivity for LSDV. (D) PCR products obtained using LSDV_WGSPP3.5 kb primer panel for the amplification of the entire LSDV genome as ~ 3.5 kb fragments. Lane M—1 kb DNA size ladder; lane 1 & lane 3—pool A PCR product of two different samples; lane 2 & lane 4—pool B PCR product of two different samples.

Samples were collected from dairy cows from Pune, Nashik, and Ahmednagar districts of Maharashtra state, and Mayurbhanj and Angul districts of Odisha state (Fig. 1A and B). The metadata details of the samples collected, including herd-level data such as geo-location and animal-related data such as age, breed, vaccination status, and history of LSDV infection, are given in Supplementary Table 1. Approval for sample collection was obtained from the animal ethics and biosafety committees of BAIF Development Research Foundation, Pune. Samples were collected in 2022 and 2023 from herds having infected animals during LSDV outbreaks following the methodology described in the protocols.io document (dx.doi.org/10.17504/protocols.io.yxmvm3drbl3p/v1). Infected animals were identified by the presence of characteristic skin nodules, which in some animals had turned into skin lesions. Nasal swab samples were collected from visibly normal animals that lacked any LSDV symptoms to assess the feasibility of detecting asymptomatic infections.

A total of 123 samples were collected non-invasively from different locations. From Maharashtra state, skin scab swabs were collected from animals with visible LSD symptoms (skin nodules and lesions) from Nashik (1 animal), Ahmednagar (18 animals), and Pune (13 animals) districts. From four symptomatic animals from Ahmednagar, both skin scab and nasal swabs were collected. From animals without LSD symptoms (no skin nodules or lesions), nasal swabs were collected from Nashik (13 animals) and Ahmednagar (28 animals) districts. In Odisha state, samples were collected from symptomatic animals only: 24 skin and 1 nasal samples from Mayurbhanj district and 19 skin and 6 nasal samples from Angul district. The animals from which nasal samples were collected in Odisha exhibited mild symptoms with small skin nodules and no visible wounds (Fig. 1A and B). DNA extraction was carried out using the MagNA Pure 96 Automation System and reagents (Roche Diagnostics). The DNA samples were first tested for LSDV positivity using a multiplexed nested PCR protocol developed in this study. Positive samples were then taken ahead for viral genome sequencing.

### Multiplexed nested PCR for detection of lumpy skin disease virus

A nested multiplex PCR method was developed for the detection of LSDV. The nested PCR primer pairs were designed based on the reference genome of LSDV (GenBank accession NC_003027.1; Lumpy Skin Disease virus NI-2490) to amplify three distinct genomic locations (Supplementary Fig. 1A). Amplification of the three regions together provides increased sensitivity and ensures PCR detection even if a partially degraded viral genome is present in the sample. The 1 kb size products of the outer primer pairs of the nested PCR will be the template for the amplification of the 500 bp PCR products by the inner primer pairs. A detailed description of the complete protocol, primer sequences, and PCR conditions is available in protocols.io (dx.doi.org/10.17504/protocols.io.yxmvm3drbl3p/v1).

To evaluate the analytical sensitivity of the nested multiplex PCR assay, the purified 1 kb fragments obtained from the outer PCRs were quantified and used for spiking 50 ng of cattle blood DNA samples at 100, 10, 1, and 0.1 fg template amounts. DNA without template spiking was used as a negative control (Supplementary Fig. 1B). Template DNA copy number estimations in respective dilutions were performed using the NEBiocalculator online tool.

### Multiplexed amplification and Oxford Nanopore technologies sequencing of lumpy skin disease virus genomic segments

Multiplex PCR amplification of genomic segments of LSDV was developed employing a previously reported methodology (Quick et al. 2017), for Zika and Severe Acute Respiratory Syndrome Coronavirus 2 (SARS-CoV-2) genome amplification and sequencing. Initially, the primer pairs reported for LSDV genome amplification (Mathijs et al. 2022) (denoted in this study as the LSDV_WGSPP7.5 kb primer panel; Supplementary Table 2) were used to test their performance in multiplexed PCR amplification of 7.5 kb overlapping fragments covering the entire LSDV genome (Supplementary Fig. 2A). Upon sequencing of the PCR products, it was observed that some of the primer pairs were not working in the multiplexed assay. Therefore, a new set of primers (denoted as the LSDV_WGSPP3.5 kb primer panel) was designed to amplify shorter 3.5 kb genomic amplicons with uniform efficiency in multiplex PCR, covering 99.8% of the LSDV genome. These primers were designed using the PrimalScheme (Quick et al. 2017) and Primer3 (Untergasser et al. 2012) tools, using conserved regions as per the LSDV genome sequences available in National Center for Biotechnology Information (NCBI) and from preliminary genome sequence data generated from field samples collected for this study. Details of LSDV genome amplification by multiplex PCR are available in protocols.io (dx.doi.org/10.17504/protocols.io.yxmvm3drbl3p/v1).

Further, different DNA polymerases, such as Quantabio repliQa HiFi ToughMix®, Takara Bio PrimeSTAR GXL Premix, NEB Q5® Hot Start High-Fidelity 2X Master Mix, and NEB LongAmp® Taq DNA Polymerase, were tested for their ability to give high-quality and uniform level of PCR products over the entire length of the LSDV genome. The PCR products were processed for library preparation for Oxford Nanopore Technologies (ONT) sequencing as given in the protocols.io document. Sequencing was done to obtain a minimum depth of 50X genome-wide coverage, and real-time assessment of sequencing depth was done using the Rampart (Artic Network. ^*^RAMPART^*^, Version 1.0.0. 2020. https://artic.network/rampart). software and reference data as mentioned in GitHub (https://github.com/ajinkyakhilari/virAssem_2).

### Lumpy skin disease virus variant identification and phylogenetic analysis

A bash script named **virAssem_2.sh** was developed for automated analysis of sequence data; this script is accessible from GitHub (https://github.com/ajinkyakhilari/virAssem_2). The initial step involved converting raw sequence read files to fastq format, followed by demultiplexing using the Guppy (Guppy Basecaller^*^, Version 6.1.5. 2024a. https://nanoporetech.com) (v6.1.5) super accurate basecalling algorithm, and then a quality control (QC) step was performed on the fastq files using the fastp (Chen et al. 2018) (v0.23.4) software. Files that passed QC criteria were mapped to the reference genome using the minimap2 (v2.27) (Li 2018) tool. Primer sequences were eliminated using the Bamclipper (v1.0.0) (Au et al. 2017) tool, and variant calling was executed in a sequential manner utilizing the medaka (v2.0.0) (Medaka^*^, Version 1.8.0. 2024b. https://github.com/nanoporetech/medaka), and longshot (v1.0.0) (Edge and Bansal 2019) variant callers. Variants that passed quality filters were then used for reference-guided assembly. This involved generating a pre-consensus FASTA through BCFtools (v1.21) (Genovese et al. 2024), followed by depth masking using Bedtools (v2.28.0) (Quinlan and Hall 2010) to mask positions with sequencing depth below 20X. In the final step, variant annotation was performed using the SnpEFF (v5.2c) (Cingolani et al. 2012) tool. A Python script was used to create nucleotide-level depth plots of the sequence data mapped to the reference genome.

Phylogenetic analysis was carried out for a total of 174 LSDV genomes: 41 from this study, 6 downloaded from GitHub repository (Bhatt et al. 2023), and 127 downloaded from GenBank (of length > 150 kb in data available up to November 2024). The accession ID list for all genomes used in this analysis is provided in Supplementary File 1. Multiple sequence alignment was done using Multiple Alignment using Fast Fourier Transform (MAFFT) (v7.525) (Katoh et al. 2002) and a maximum likelihood phylogenetic tree was constructed using IQ-TREE2 (Minh et al. 2020). ModelFinder Plus was used to automatically determine the best-fit substitution model for sequence alignment. The corresponding rooted and unrooted phylogenetic trees were rendered using Interactive Tree Of Life (ITOL) (Letunic and Bork 2021). The phylogenomic data was inferred based on the LSDV clades described previously (Breman et al. 2023).

## Results

### Evaluation of the nested multiplex PCR for detection of lumpy skin disease virus

The performance of the multiplexed nested PCR assay for detection of the virus was validated with samples collected from animals that were LSD positive with visible skin lumps and lesions (Fig. 1C). The specificity of the nested PCR assay was established by the lack of non-specific amplification from cattle DNA. The detection of as low as 0.1 fg of viral DNA (equivalent to DNA from 30 viral particles) in a background of 50 ng of cattle DNA showed that the multiplexed nested PCR based LSDV detection was highly sensitive (Supplementary Fig. 1B). The presence of the LSDV in field samples was confirmed by this PCR assay before viral genome amplification and sequencing.

### Field sample collection and lumpy skin disease virus positivity

**Figure 2:**
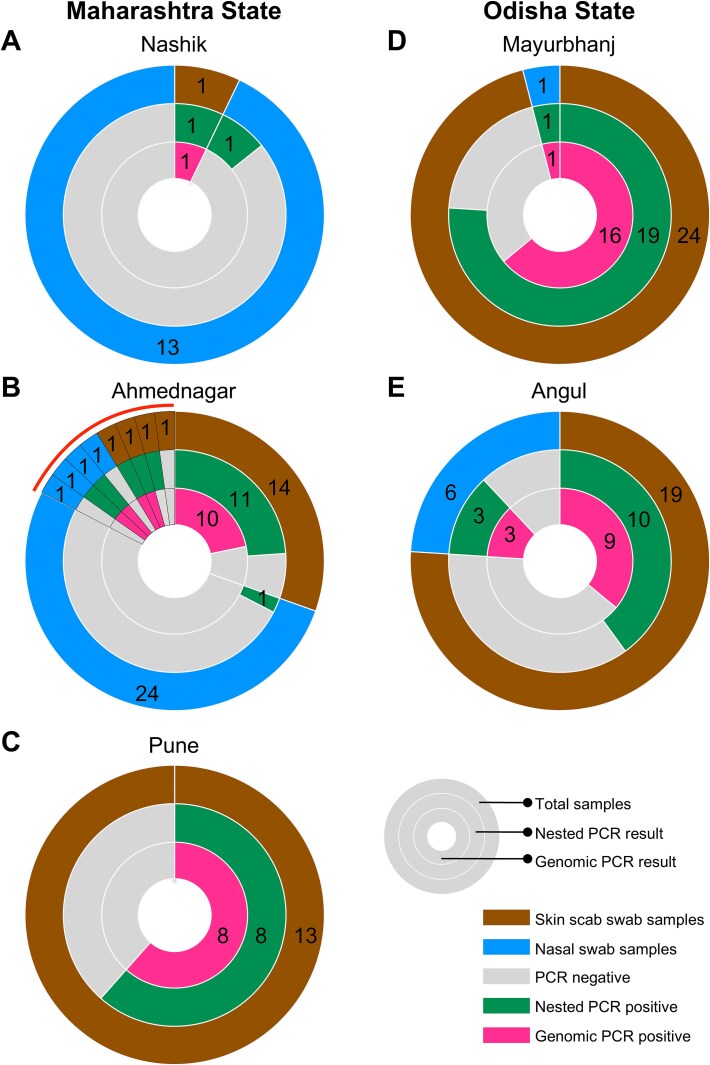
Number of samples collected and analysis outcome: the plots illustrate the sample numbers for which nested PCR-based virus detection was successful and genome sequencing of LSDV was achieved. (A–C) Samples from Maharashtra state; (D and E) samples from Odisha state. The red line in the Ahmednagar plot highlights the four pairs of skin scabs and nasal swab samples collected from four animals, shown in order.

A first set of skin scab samples was collected in 2022 from animals that were symptomatic for LSDV infection with skin lesions from the Pune district of Maharashtra, India. In 2023, samples were collected from the Pune, Nashik, and Ahmednagar districts of Maharashtra state and the Mayurbhanj and Angul districts of Odisha state (Fig. 1B). In addition to the collection of skin scab samples from symptomatic animals, nasal swab samples were also collected from both symptomatic and asymptomatic animals. LSDV has been detected from nasal mucosal swabs (Bhatt et al. 2023, Reddy et al. 2023) and can be a useful method for surveillance and to identify animals with asymptomatic infection. Details of the total samples collected and the number that tested positive are given in Fig. 2.

Out of the 123 samples that were analysed, nested PCR positivity was obtained for 60 samples (Fig. 2). In symptomatic animals from Maharashtra, 23 out of 32 skin and 2 out of 4 nasal samples were positive. In nasal samples collected from animals without symptoms, 2 out of 37 were positive (Fig. 2A–C). In symptomatic animals from Odisha, 29 out of 43 skin and 4 out of 7 nasal samples were positive (Fig. 2D and 2E). The metadata details for all samples, along with LSDV detection and genome sequencing outcomes, are given in Supplementary Table 1.

### Lumpy skin disease virus genome sequencing and genetic variation analysis

Whole LSDV genome amplification was successfully achieved in 52 out of 60 samples that were nested PCR positive for the virus (Fig. 2). Initially, for six samples collected in 2022 from the Pune district, genome amplification was carried out using the LSDV_WGSPP7.5 kb primer panel. However, from the sequence data, it was apparent that a few of the target regions were sub-optimally amplified and did not yield good sequence data. For these regions, individual PCR amplicons were obtained and sequenced. Despite these technical issues, the amplicons obtained from six samples using the LSDV_WGSPP7.5 kb primer panel were sequenced by ONT with sufficient quality and read depth to allow variant calling and genome assembly (Supplementary Fig. 2B).

**Figure 3:**
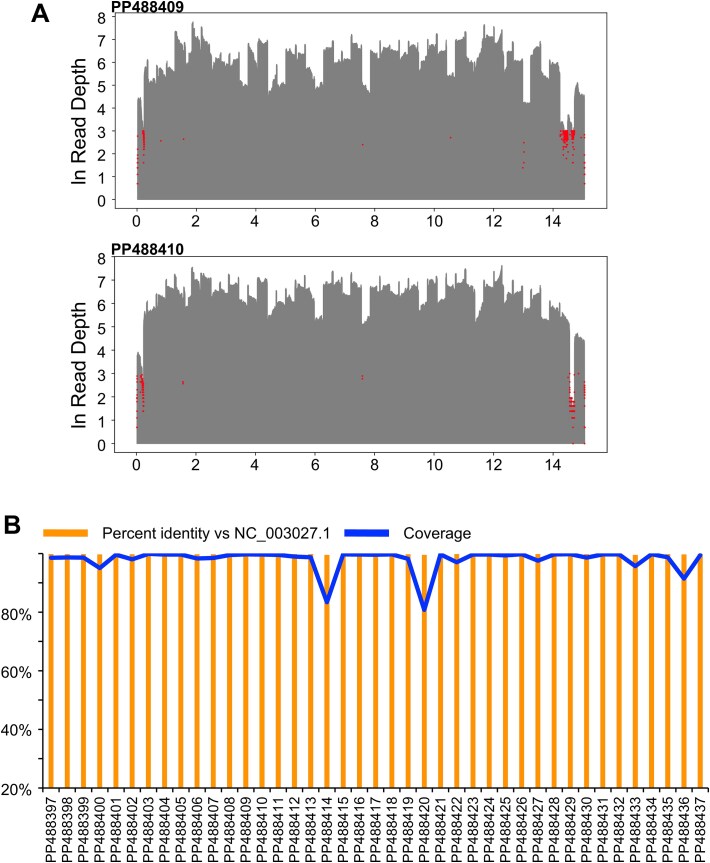
LSDV genome coverage by Nanopore sequencing: (A) Nucleotide level read depth plots of two representative LSDV genomes obtained by Nanopore sequencing of multiplexed genomic PCR amplicons generated using LSDV_WGSPP3.5 kb primer panel (red marks indicate the positions at which the read depth was <20X). (B) Sequence coverage (blue) and percent identity (orange) plots for the 41 field isolate genomes in comparison with the reference sequence NC_003027.1.

To make the genome amplification step more robust, the LSDV_WGSPP3.5 kb primer panel was designed and demonstrated to work efficiently in multiplexed PCR conditions (Fig. 1D). The genomic amplicons obtained from multiplexed PCR with this primer panel, consisting of 48 overlapping fragments of ~3.5 kb size covering the entire genome, showed improved sequencing coverage and depth (Fig. 3A) compared to the sequence data obtained with the amplicons generated by the LSDV_WGSPP7.5 kb primer panel (Supplementary Fig. 4A and B). Except for the initial six samples, LSDV genome amplification of all other samples was carried out using the LSDV_WGSPP3.5 kb primer panel.

It was also observed that the optimal performance of the multiplexed PCR and quality of the amplicons were influenced by the type of Taq DNA polymerase used. In this study, different Taq DNA polymerases were tested, such as Quantabio repliQa HiFi ToughMix® polymerase, Takara Bio PrimeSTAR GXL Premix, NEB Q5® Hot Start High-Fidelity 2X Master Mix, and NEB LongAmp® Taq DNA Polymerase (Supplementary Fig. 3A and B). Of these, the best PCR performance and good quality Nanopore sequence data were obtained with the Quantabio repliQa HiFi ToughMix® polymerase (Fig. 3A). The performance of Takara Bio PrimeSTAR reagent was inconsistent across samples (Supplementary Fig. 4C and D) and NEB Q5® and LongAmp® reagents did not support multiplexed PCR amplification.

**Figure 4:**
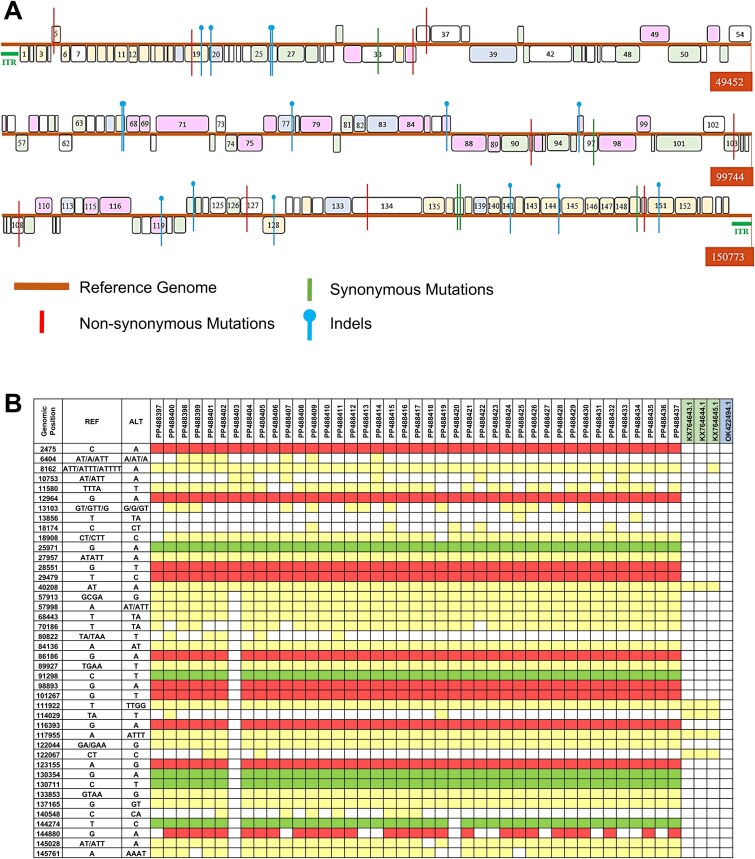
Mutation profile of LSDV from field samples: (A) Schematic representation of the LSDV reference genome showing the location of all 156 genes. The genomic position in base pair is shown within a red box at the end of each line and each gene is shown as a coloured box (width scaled relative to gene size in bp), and the numbers shown inside the gene boxes indicate the order in which the genes occur. Each gene is coloured by function, and the position of the genes above or below the horizontal line indicates the forward and reverse direction of coding. ITRs are shown as green bars at the two ends of the linear genome. Novel mutations are shown as vertical lines and are mapped to genomic position; green—synonymous mutation; red—non-synonymous mutation; blue—indels. Gene function colour code: pink—RNA transcription and modification (26 genes); yellow—viral virulence and host range (37 genes); green—structure and assembly (33 genes); blue—DNA replication and nucleotide metabolism (10 genes); white—unknown function (50 genes). (B) Co-occurrence of novel mutations in the field strain LSDV genomes. Position-wise list of all novel mutations identified from the LSDV genomes sequenced from 41 field samples analysed in this work is given. Column headers: REF—reference genotype (NC_003027.1); ALT—mutant genotype seen in field samples. Mutation colour code: red—non-synonymous; green—synonymous; yellow—indels; white—same as reference. The highlighted accession IDs indicate the African Neethling vaccine strains (green) and the attenuated strain developed as LSDV vaccine in India (blue).

Out of the 52 field samples for which LSDV genome sequencing was carried out, based on genome coverage data (>80% at least), only 41 samples were selected for further analysis. The percent identity of these 41 LSDV genomes with the reference LSDV genome (NC_003027.1) was > 99% (Fig. 3B). Genetic variations in the virus from field samples were identified using NC_003027.1 as the reference genome, as described in the GitHub repository. These variations ranged from 98 to 271 distinct changes per individual genome (Supplementary File 2). Most of these variations are also present in 133 LSDV genomes reported prior to 2022 (Supplementary Fig. 5). Only 41 mutations appear to be of recent origin and can be seen in LSDV sequenced from India from 2022 onwards (Fig. 4).

Other reports on LSDV genome sequencing from India have identified a similar set of mutations (Bhatt et al. 2023) suggesting the emergence and spread of a novel LSDV genotype in India in recent years. Interestingly, 6 novel mutations (C6621A, G8023A, G8025T, T16016TA, A30663T, and T121486A) originally reported in samples from Rajasthan in 2022 (Bhatt et al. 2023) are missing in the samples analysed in this study. This reveals that there are likely multiple sub-lineages of LSDV infecting dairy cattle. Of the 41 mutations, 10 are non-synonymous, 5 are synonymous, and 26 are indels distributed in coding and non-coding regions (Table 1). Six out of 10 non-synonymous mutations were present in genes involved in viral virulence and host range, RNA transcription and modification, and structure and assembly (Table 1, Fig. 4). These functions are important for viral replication and virulence, and amino acid changes in the corresponding proteins can potentially affect disease outcome.

Three genes encoding viral virulence factors, *viz* kelch-like protein (LSDV019), putative host range protein (LSDV067), and a gene with unknown function (LSDV026), are affected by multiple mutations. The indels detected in the coding region of three genes (position 13 103 in LSDV019, position 18 174 in LSDV026, and position 80 822 in LSDV087) result in truncation of the coding sequence and likely result in disruption of gene functions. Indels in three other genes (LSDV077, LSDV122, and LSDV144) occur at or near the stop codon and may have minimal or no effect on protein function. Notably, 22 of the 41 identified mutations were present in 40 out of 41 LSDV genomes reported in this study, indicating their widespread occurrence and dominance among circulating strains. In general, high-frequency mutations (detected in 40 genomes) were predominantly located within coding regions, whereas the lower-frequency mutations were mainly observed in non-coding regions (Table 1).

### Phylogenomics and evolution of lumpy skin disease virus

Phylogenetic analysis was carried out for all available LSDV genomes (41 from this study and 133 available from NCBI GenBank database and GitHub repository). The sequences were aligned, and the IQTREE2 maximum likelihood algorithm was used to construct the phylogenetic tree. Phylogenetic analysis was performed using IQ-TREE with ModelFinder to select the best-fit substitution model. The K3Pu + F + I + R5 model was chosen as optimal according to the Bayesian Information Criterion. A detailed description of this model can be found at https://iqtree.github.io/doc/Substitution-Models.

**Figure 5:**
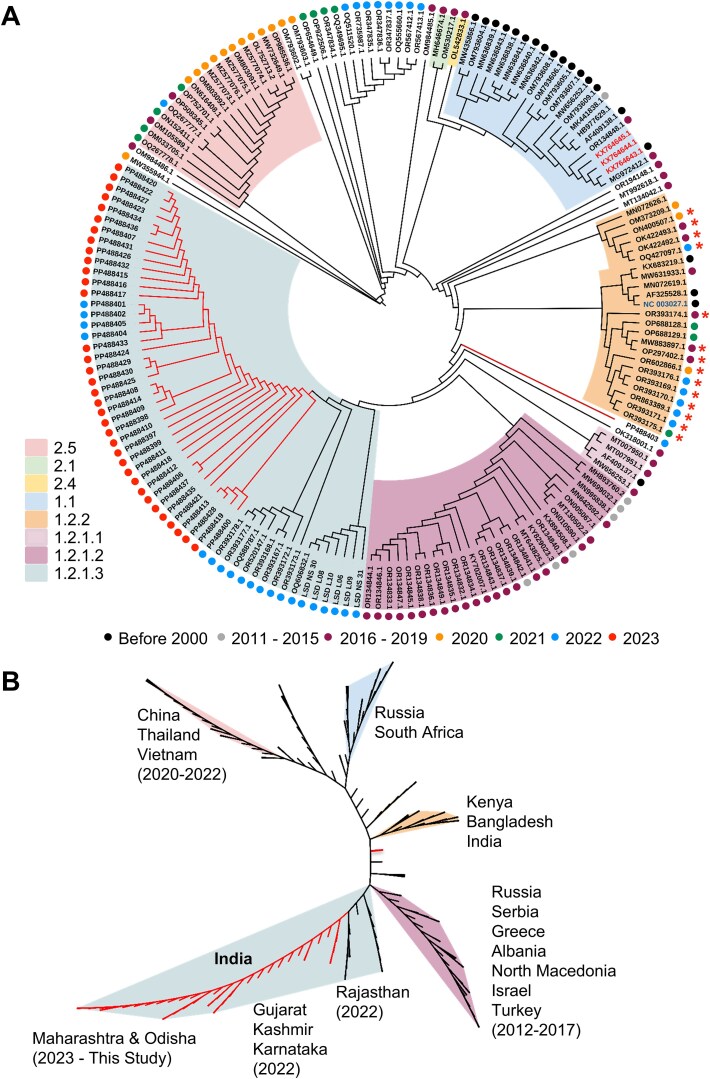
Phylogenomic relationship of LSDV field strains with previously reported isolates: Maximum likelihood phylogeny was performed for the LSDV genomes sequenced in this study (41 field samples) and 133 other global LSDV genomes and the resulting tree file was used to render rooted (A) and unrooted (B) tree structures. (A) rooted radial tree annotated with NCBI accession ID of each LSDV genome and coloured dots next to the sample name to indicate the year of isolation/sampling of the virus from the field. The sequences reported from India, which are grouped in clade 1.2.2, are indicated with a red asterisk and the accession IDs of the African Neethling vaccine strains are shown in red font, while the reference strain (NC_003027.1) is shown in blue font. (B) In the unrooted tree, the geographic region from which the viruses were reported is shown.

It is apparent from the phylogeny that LSDV strains from India, sampled in 2022 and 2023, form two distinct clades. A small number of LSDV strains from India sampled in 2022 grouped within clade 1.2.2 which also includes the Neethling 2490 strain (NC_003027.1; used as reference for variant identification), which was originally isolated from Kenya in 1959 (Mazloum et al. 2023), 2 Bangladesh strains sampled prior in 2021 and other strains from India sampled between 2016 and 2019 (Fig. 5A and B). However, most of the LSDV strains sampled from India in 2022 and 2023 (including all except one (PP488403) reported in this study), grouped in clade 1.2.1.3 and are closer to LSDV strains from Russia, Serbia, and Eastern European countries sampled between 2011 and 2019 (clade 1.2.1.2) (Fig. 5A). The PP488403 genome has a chimeric profile of novel mutations detected in other viruses sequenced in this study (Fig. 4B), and groups along with the older genotypes of the virus from India, Bangladesh, and Kenya (clade 1.2.2). The LSDV strain genomes reported from China, Thailand, and Vietnam between 2016 and 2022 formed a distant clade (clade 2.5) and are closer to viruses grouping in clade 1.1 (Fig. 5A and B).

When the genome-wide genetic variations between viruses from different clades were compared (Supplementary Fig. 5), it was apparent that clades 2.5, 2.1, 2.4, and 1.1 exhibited a larger number of variations when compared to the reference Neethling strain (clade 1.2.2). Clades 2.5 and 2.4, while genetically distinct, are proximal in phylogeny, highlighting their genomic relatedness compared to the more divergent clade 1.1. On the other hand, all viral genomes grouped within clade 1.2 (i.e. sub-clades 1.2.1.1, 1.2.1.2, 1.2.1.3, 1.2.1.1, and 1.2.2) have comparatively fewer variations, which are largely conserved. It should be noted that there is a clear distinction between clades 1.1 and 1.2 (and its sub-clades) in genetic similarity with the LSDV reference genome used to identify the variations. As a result of the small number of distinct variations seen in the LSDV genomes reported from India in this and previous studies (Bhatt et al. 2023, Yadav et al. 2024) they form a distinct clade which has been named as clade 1.2.1.3. The genomes of the attenuated strains used for developing the LSDV vaccine in India (Kumar et al. 2023) are grouped in clade 1.2.2.

**Table 1:** List of novel mutations identified from LSDV field strains from India sampled during 2022–23

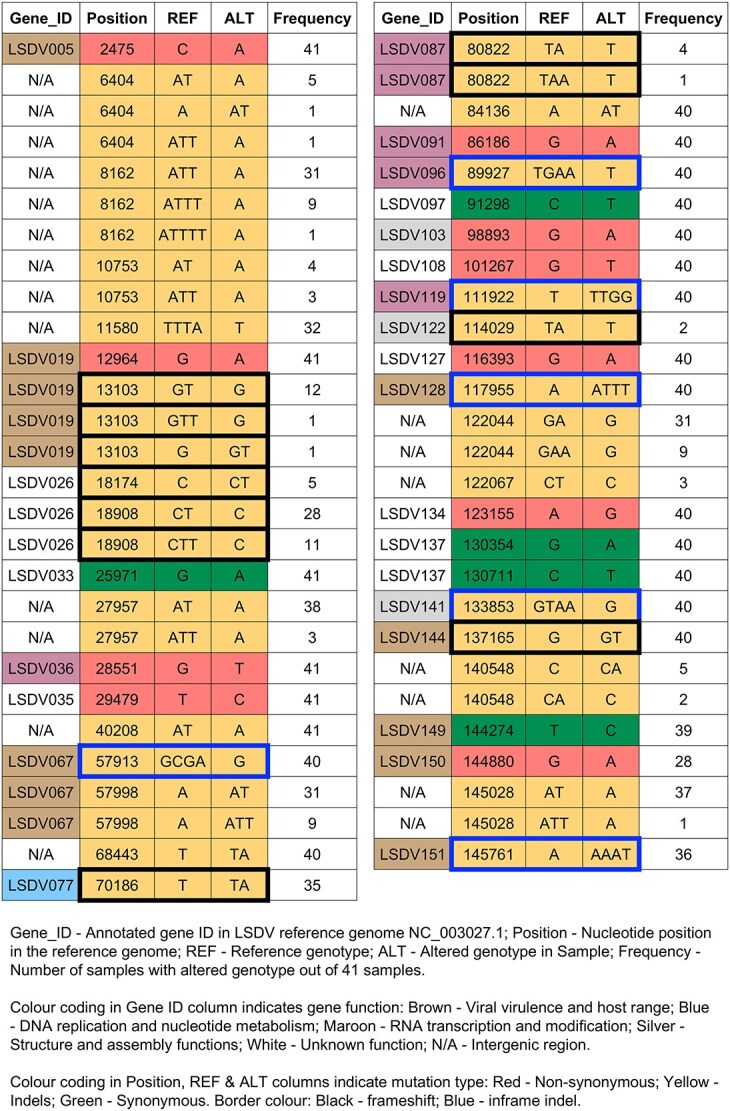

The genotype of the mutations, along with the genomic positions and genes affected, are tabulated. The frequency column indicates the number of samples in which the mutation was detected, in a total of 41 samples.

## Discussion

The recent emergence and spread of LSD among dairy cattle in India has had devastating consequences on animal health, mortality, and dairy productivity. Therefore, it is important to understand the relevance, transmission, host-virus interaction and disease outcome for LSD. Current preventive measure relies solely on vaccination and in India the goat pox vaccine, known to give partial protection against LSD (Reddy et al. 2023) was authorized for use as a heterologous vaccine. A new live-attenuated LSD vaccine (Lumpi-ProVac^Ind^), with high efficacy and safety profiles, developed using a 2019 isolate from Ranchi, India, appears to be protective against LSDV (Kumar et al. 2023). A recent study has identified clade 2.5-like LSDV virus in the Great Nicobar Island in India, suggesting the spread of this clade of virus from Southeast Asia (Sudhakar et al. 2025). Therefore, continuous monitoring of the LSDV genotypes circulating in the field is needed to keep track of genetic variations that may affect vaccine efficacy or alter disease severity. The possibility of LSDV affecting other animals also exists, as reported from camels and gazelles (Mazloum et al. 2023, Sudhakar et al. 2023) and expanded host range can also potentially result in genomic alterations. The close contact between LSDV-infected cattle and dairy farmers warrants the necessity to monitor this disease from a zoonotic perspective as well. In this study, an easy-to-use and scalable method for the detection and genotyping of LSDV from dairy cattle was developed and validated with field samples.

For the detection of the virus, a multiplexed nested PCR assay was developed. Three different highly conserved regions were chosen for PCR amplification using the genome sequence of the reference LSDV (NC_003027.1) and other LSDV isolates available from NCBI (Supplementary Fig. 1A). Since the different pox viruses belonging to capripoxvirus group are highly conserved, the sequences selected for this PCR assay were conserved in goat and sheep pox viruses as well. This provides the feasibility of detecting multiple capripoxviruses in a single assay by sequencing the PCR products. The three nested PCRs were optimized to work in a single multiplexed reaction and were demonstrated to have very high sensitivity (robust detection even at ~30 copies of the virus in the assay; Supplementary Fig. 1B). With this level of sensitivity, detection of LSDV from asymptomatic cattle using non-invasive sample collection (such as nasal swabs) is possible. Field samples collected from the states of Maharashtra and Odisha were tested for LSDV detection using the multiplexed nested PCR. In addition to detecting the virus from skin lesion swabs, the virus was also detected from nasal swabs, which is useful for testing animals with mild symptoms or asymptomatic ones (Fig. 2). This method is designed for quick and reliable virus detection, and can be valuable for LSD surveillance, especially during seasonal outbreaks, and monitoring vaccine efficacy.

To study the genetic variations in the LSDV strains circulating in India and to understand the phylogeny and evolution of the virus, LSDV genome sequencing was carried out using Oxford Nanopore sequencing. Whole genome amplification using a panel of primers amplifying ~ 3.5 kb regions (designated as the LSDV_WGSPP3.5 kb) was designed and validated (Figs 1D and 3). Using this method, the LSDV genome sequence (with >80% coverage) was generated from 41 samples (6 samples from 2022 and 35 samples from 2023). While all 41 genomes had >99% identity with the reference genome (NC_003027.1), many changes (ranging from 98 to 271 variations per genome; Supplementary File 2) were also detected. While most of these variations can be seen in previously reported genomes, a number of novel mutations conserved across the LSDV genomes from field samples were detected (Table 1 and Fig. 4B). These include 10 non-synonymous, 5 synonymous and 19 indels (in coding regions). Indels in LSDV019, LSDV026 and LSDV087 genes disrupted the respective coding sequences, potentially resulting in loss of function. Nine commonly occurring mutations were enriched in the genes with viral virulence and host range function (LSDV005, LSDV019, LSDV067, LSDV128, LSDV144, LSDV150, LSDV151). From the pattern of shared genetic variation between the different viral genomes, it is apparent that the PP488403 virus has a distinct profile. The 4 non-synonymous and 1 synonymous mutation found in PP488403 located in genomic positions 2475, 12 964, 25 971, 28 551, and 29 479 are highly conserved and found in all the genomes. However, all downstream mutations are absent in the PP488403 virus, which suggests that multiple sub-lineages of LSDV with subtle genetic differences may be circulating in India. The higher proportion of mutations in virulence and replication-associated genes may reflect selective pressures on LSDV to adapt to new hosts or environmental conditions. This pattern, also noted in previous Indian isolates (Bhatt et al. 2023, Yadav et al. 2024) supports the possibility of ongoing diversification of viral sub-lineages.

The Indian LSDV strain genomes characterized in this study (clade 1.2.1.3) were found to be more closely related to the LSDV genomes reported from Russia, Serbia and other Eastern European and Central Asian countries (clade 1.2.1.2) (Fig. 5B). Due to the absence of more recent LSDV genome sequences in these regions for the 2022–23 period, it is unclear if the LSDV genotypes detected in India are also currently circulating in these regions. While LSDV has undergone significant genetic changes and evolved in recent years, the origin and factors driving these changes are not readily apparent. The high level of conservation of the novel mutations in the Indian LSDV isolates analysed in this and other (Bhatt et al. 2023, Yadav et al. 2024) studies suggest a common origin and spread of this genotype of the virus. Previous studies have identified two distinct LSDV genotypes circulating in India (clade 1.2.2 and clade 1.2.1.3) based on analysis of samples collected up to 2022 (Breman et al. 2023) (Fig. 5). All, except one (PP488403), LSDV genomes generated in this study grouped in clade 1.2.1.3.

In India, the currently used vaccine is the heterologous goat pox vaccine, while in African countries vaccines are derived from the Neethling strain of LSDV. Importantly, none of the India-specific mutations identified in this study are present in the African Neethling vaccine strains (Fig. 4B). The LSDV genotypes characterized from India reveal the need for monitoring this virus from field samples in the coming years to track new genotypes and correlate with the disease severity and vaccine efficacy. The detection and genotyping methods described in this work address this requirement and can be scaled up for country-wide surveillance of LSDV in dairy cattle.

## Supplementary Material

Bajpai_et_al_Supplementary_File_1_veaf090

Bajpai_et_al_Supplementary_File_2_veaf090

Bajpai_et_al_Supplementary_Table_1_veaf090

Bajpai_et_al_Supplementary_Table_2_veaf090

Bajpai_et_al_Supplementary_Figure_1_veaf090

Bajpai_et_al_Supplementary_Figure_2_veaf090

Bajpai_et_al_Supplementary_Figure_3_veaf090

Bajpai_et_al_Supplementary_Figure_4_veaf090

Bajpai_et_al_Supplementary_Figure_5_veaf090

Supplementary_captions_veaf090

Bajpai_et_al_Supplementary_Table_2_veaf090

## Data Availability

LSDV genome sequences data generated from this study has been deposited in GenBank under the bio project ID PRJNA1086146.

## Software availability

Data analysis software and pipeline used in this study can be accessed from GitHub using this link: https://github.com/ajinkyakhilari/virAssem_2.

## Protocol availability

A detailed step-by-step protocol document can be accessed from Protocols.io repository using this link: dx.doi.org/10.17504/protocols.io.yxmvm3drbl3p/v1.
