# Supplementary figures and images for: Detection and variant characterization of lumpy skin disease virus from dairy cattle in India
Source: Virus Evol. 2025 Nov 20;11(1):veaf090. doi: 10.1093/ve/veaf090 (PMC12678169; doi:10.1093/ve/veaf090)

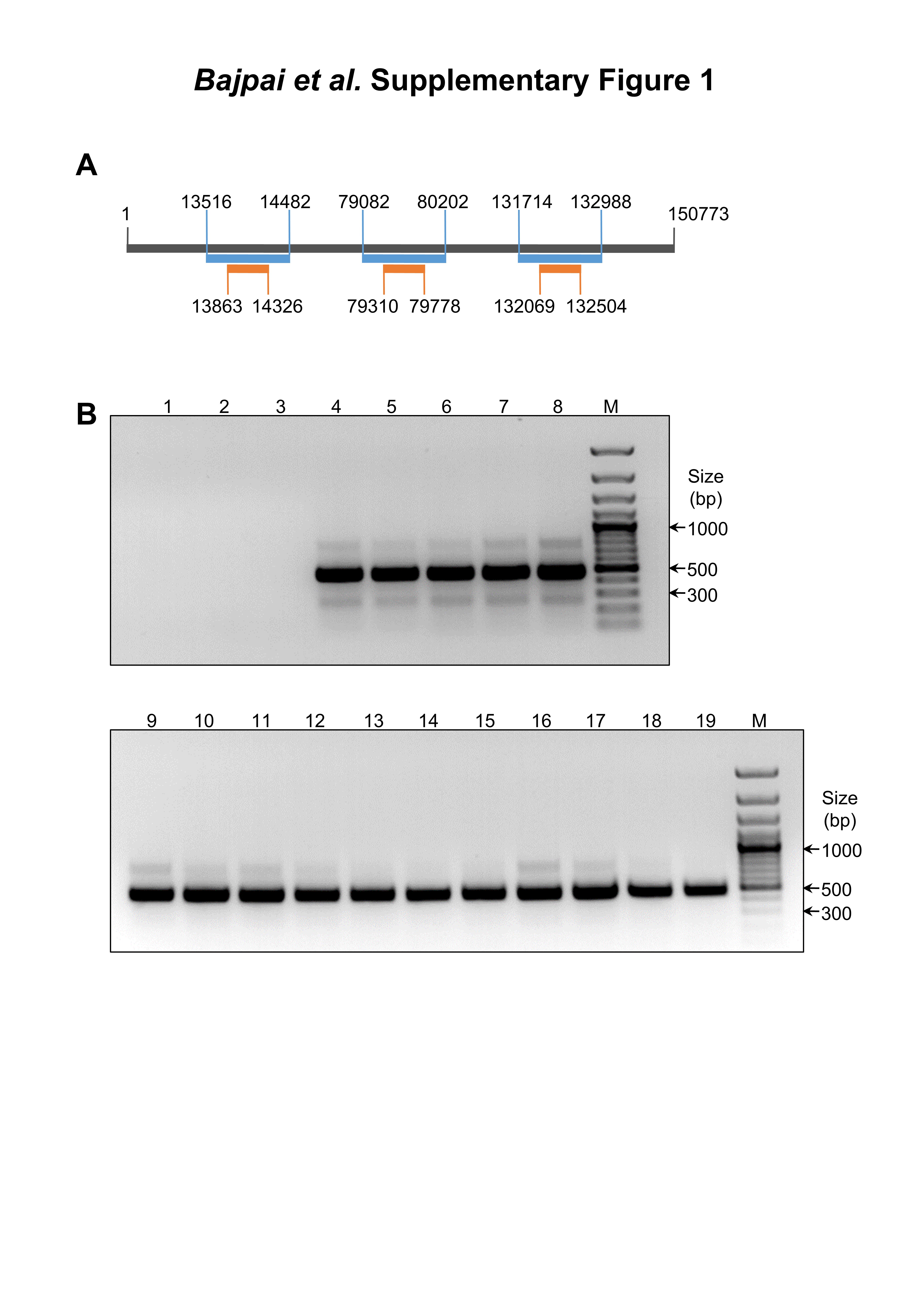

Supplement: Bajpai_et_al_Supplementary_Figure_1_veaf090 [file bajpai_et_al_supplementary_figure_1_veaf090.jpeg]

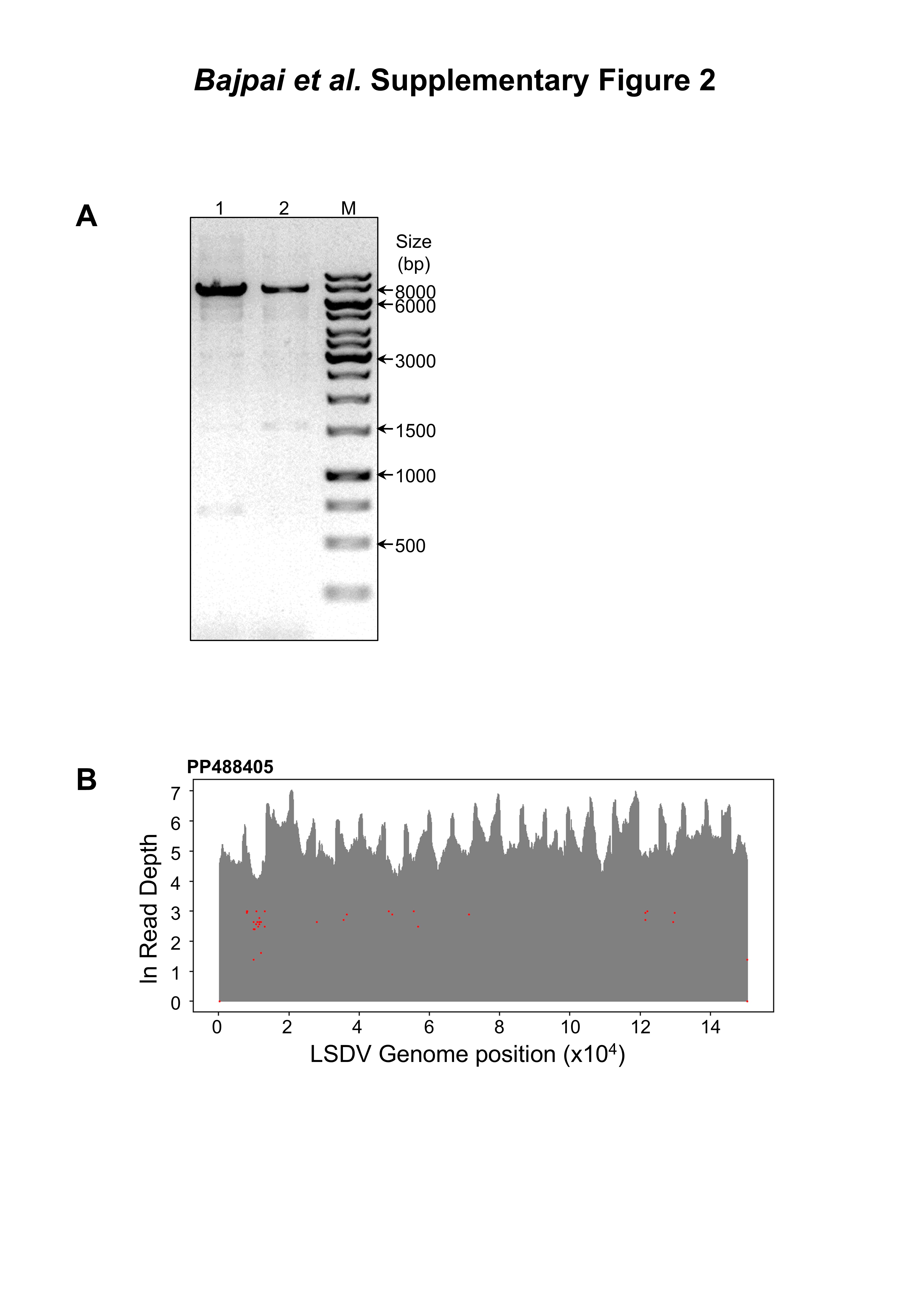

Supplement: Bajpai_et_al_Supplementary_Figure_2_veaf090 [file bajpai_et_al_supplementary_figure_2_veaf090.jpeg]

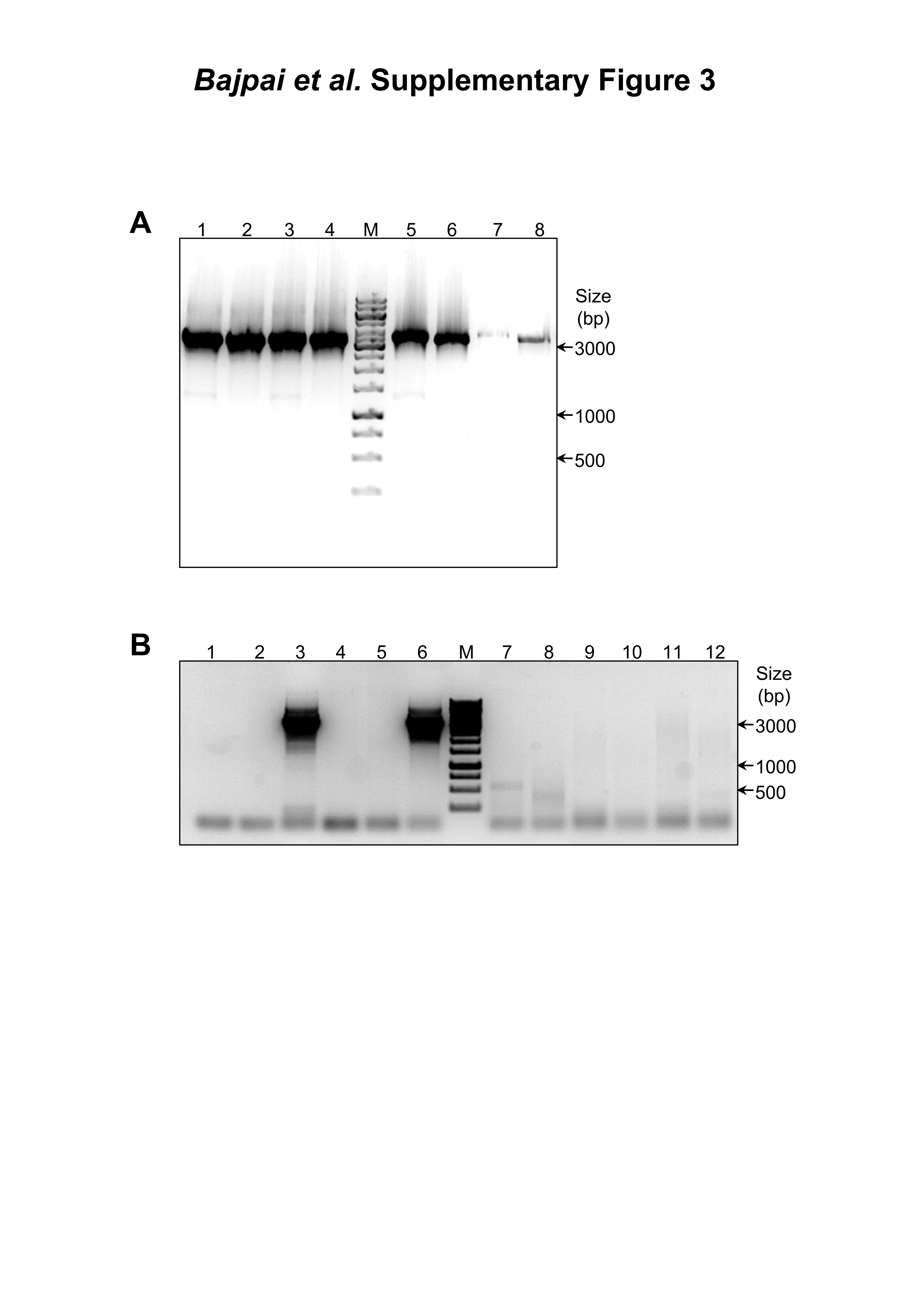

Supplement: Bajpai_et_al_Supplementary_Figure_3_veaf090 [file bajpai_et_al_supplementary_figure_3_veaf090.jpeg]

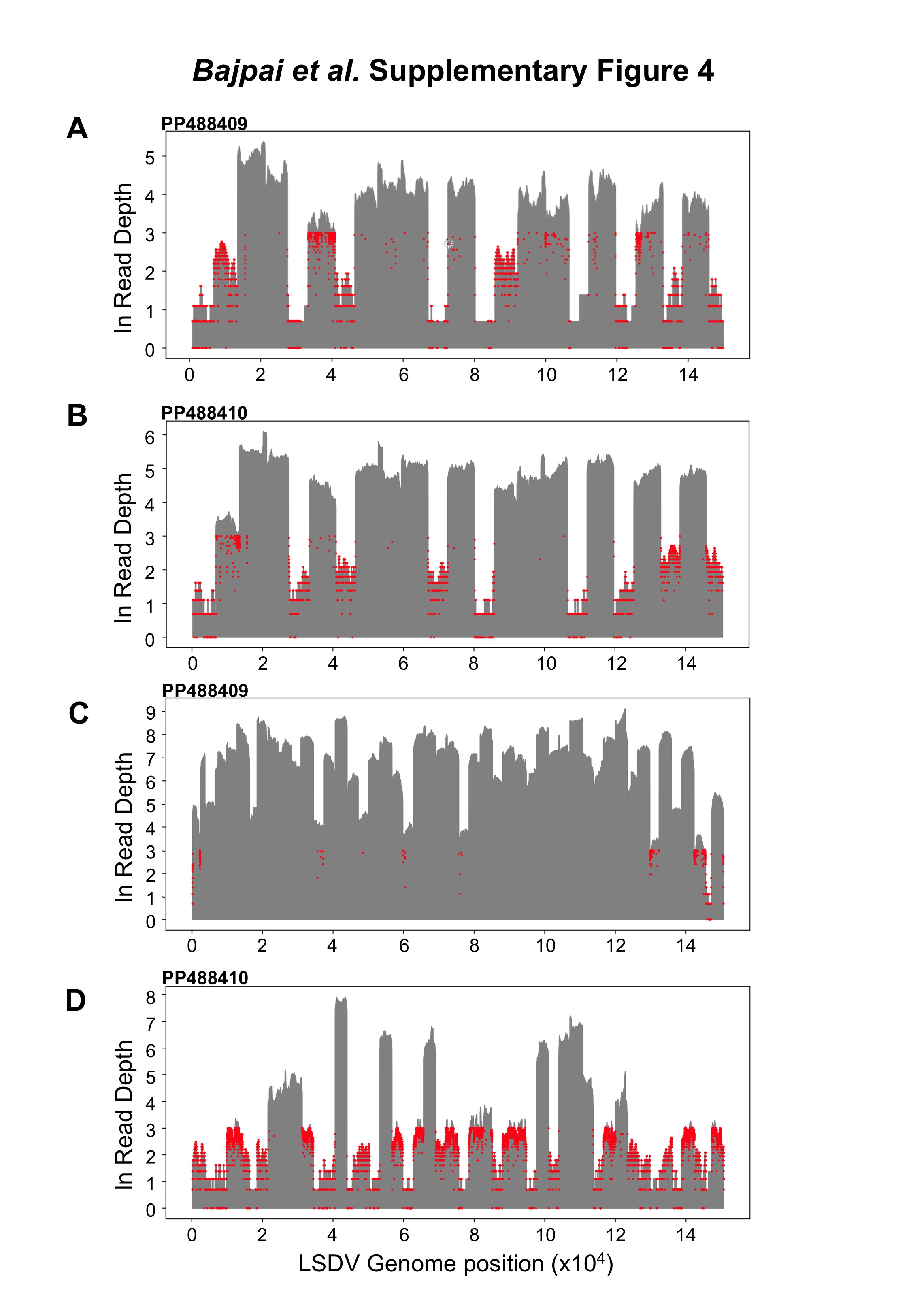

Supplement: Bajpai_et_al_Supplementary_Figure_4_veaf090 [file bajpai_et_al_supplementary_figure_4_veaf090.jpeg]

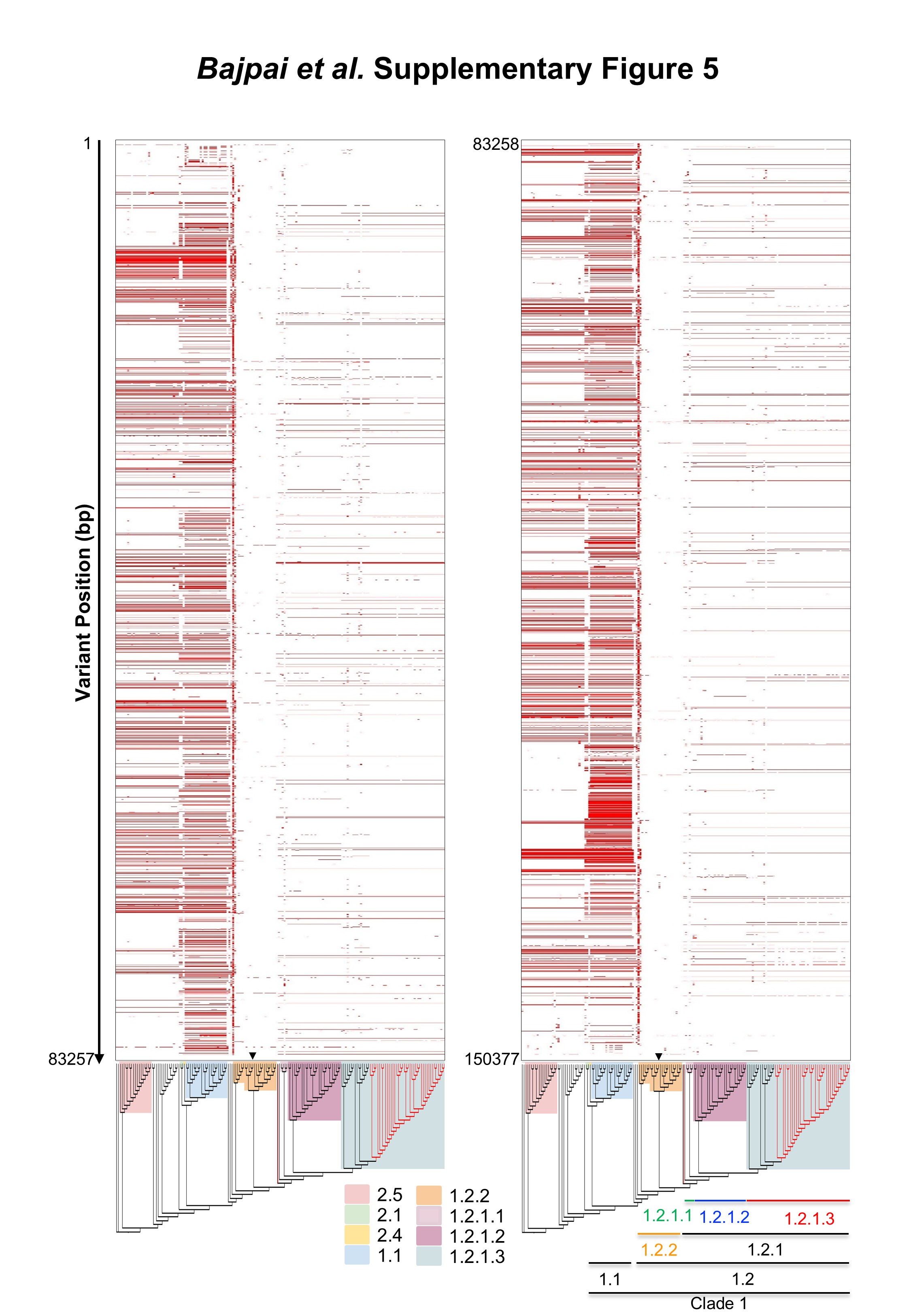

Supplement: Bajpai_et_al_Supplementary_Figure_5_veaf090 [file bajpai_et_al_supplementary_figure_5_veaf090.jpeg]
