# Supplementary material for: Detection and variant characterization of lumpy skin disease virus from dairy cattle in India
Source: Virus Evol. 2025 Nov 20;11(1):veaf090. doi: 10.1093/ve/veaf090 (PMC12678169; doi:10.1093/ve/veaf090)
Supplement: Supplementary_captions_veaf090 [file supplementary_captions_veaf090.docx]

**Supplementary Figure 1: Multiplexed nested PCR details**. ***A***, schematic depiction of the LSDV genomic coordinates for the target region of the three nested PCRs. ***B***, PCR assays showing the analytical sensitivity of the multiplexed nested PCR. Lanes 1 - 3, three different negative field samples (a, b & c); Lanes 4 - 6, samples a, b & c spiked with 100 fg of 1 kb template; Lanes 7 - 9, samples a, b & c spiked with 10 fg of 1 kb template; Lanes 10 - 12, samples a, b & c spiked with 1 fg of 1 kb template; Lanes 13 - 15, samples a, b & c spiked with 0.1 fg of 1 kb template; Lanes 16 - 19, four field samples which are positive for LSDV showing the expected 500 bp multiplexed nested PCR product. Lane M - 100 bp DNA size ladder.

**Supplementary Figure 2: LSDV genome amplification and nanopore sequencing using the LSDV_WGSPP7.5kb primer panel**. ***A***, Agarose gel electrophoresis confirmation of the ~7.5 kb PCR product obtained from two different field samples (Lane 1 & 2) collected from animals with a clinical diagnosis for LSD. Total DNA isolated from skin scab swab samples was used for PCR amplification. Lane M - 1 kb DNA size ladder. ***B***, Nanopore sequence read count plot for representative sample PP488405, for which the genome amplification was carried out using the LSDV_WGSPP7.5kb primer panel. The data plotted is at the nucleotide level, and red marks indicate the positions at which the read depth was <20X.

**Supplementary Figure 3: Comparative performance of Taq DNA polymerase enzymes for multiplexed PCR amplification of the LSDV genome**. ***A***, performance of Quantabio repliQa HiFi ToughMix^®^(Lane 1 - 4) and Takara Bio PrimeSTAR GXL Premix (Lane 5 - 8) enzymes for LSDV genome amplification in multiplexed PCR reactions using the LSDV_WGSPP3.5kb primer panel. Two field samples were used, and for each sample, pool A (Lane 1, 3, 5 & 7) and pool B (Lane 2, 4, 6 & 8) reactions were run (see dx.doi.org/10.17504/protocols.io.yxmvm3drbl3p/v1). Lane M - 1 kb DNA size ladder. ***B***, performance of NEB LongAmp^®^ Taq DNA Polymerase (Lane 1 - 6), and NEB Q5^®^ Hot Start High-Fidelity 2X Master Mix (Lane 7 – 12) for LSDV genome amplification using the LSDV_WGSPP3.5kb primer panel. Three field samples were used and for each sample pool A and pool B reactions were run (see methods section for details): Lanes 1 & 7 - pool A reaction of sample a; Lanes 2 & 8 - pool B reaction of sample a; Lanes 3 & 9 - pool A reaction of sample b; Lanes 4 & 10 - pool B reaction of sample b. Lanes 5 & 11 - pool A reaction of sample c; Lanes 6 & 12 - pool B reaction of sample c. Lane M - 1 kb DNA size ladder.

**Supplementary Figure 4: LSDV genome coverage and quality of nanopore sequence data.** Read Depth plots for nanopore sequence data of LSDV genomic fragments obtained by multiplexed amplification using two different primer panels and two different PCR enzymes. The LSDV genome was amplified by multiplexed PCR using two different primer panels, LSDV_WGSPP7.5kb (***A*** ***&*** ***B***) and LSDV_WGSPP3.5kb (***C &*** ***D***). Samples PP488409 (***A*** ***&*** ***C***) and PP488410 (***B*** ***&*** ***D***) were evaluated, using two different Taq DNA polymerases, Quantabio repliQa HiFi ToughMix^®^ (***A*** ***& B*** ) and Takara Bio PrimeSTAR GXL Premix (***C*** ***&*** ***D***), for PCR amplification. The data plotted is at the nucleotide level, and red marks indicate the positions at which the read depth in log scale was <20X.

**Supplementary Figure 5: Comparative profile of genetic variation in the LSDV genome of viruses from different clades.** Heatmap representation of single-nucleotide variants (SNVs) and INDELs in all of the 174 samples with respect to the LSDV reference genome (NC_003027.1). Genomic positions are split into two panels (1 – 83257 bp on the left; 83258 – 150377 bp on the right). In each panel, rows correspond to genomic positions and columns to individual viral isolates. A red cell denotes a mutation with respect to the reference genome at that position; a white cell denotes identical nucleotides, depicting no mutation. Beneath each heatmap is a hierarchical-clustering dendrogram, with leaf labels color-coded by clade (2.5 – pink; 2.1 – green; 2.4 – yellow; 1.1 – light blue; 1.2.2 – orange; 1.2.2.1 – purple; 1.2.1.2 – mauve; 1.2.1.3 – teal).

**Supplementary Table 1: Metadata details for 123 samples collected from dairy cattle**. The LSDV genome sequence obtained for 41 samples was deposited in GenBank, and the accession IDs are mapped. Out of 123 total samples, nested PCR for detection of LSDV was successful in 60 samples (LSDV_NMDPP PCR Result column light green shading), of which for 52 samples the multiplexed PCR products for genome amplification was obtained and sequenced (LSDV_WGSPP3.5kb PCR Result column light blue shading). Based on sequence quality and completeness of genome, 41 genome sequences were submitted to GenBank (GenBank_ID given in column with light blue shading).

**Supplementary Table 2: Details of the primers used in the LSDV_WGSPP_7.5 primer panel**.

**Supplementary File 1: List of 174 genomes used for phylogenomic analysis along with their accession IDs and year of sampling.** This information is related to Figure 5.

**Supplementary File 2: Mutation profile of 174 genomes used for phylogenomic analysis.** Mutations were identified relative to the NCBI reference genome (NC_003027.1) and include synonymous, non-synonymous, and insertion-deletion (indel) variations. Clade branches are color-coded, with major clades labeled in accordance with previously published classifications^40^.
